# Surface-Enhanced Raman Spectroscopy in Cancer Diagnosis, Prognosis and Monitoring

**DOI:** 10.3390/cancers11060748

**Published:** 2019-05-29

**Authors:** Luca Guerrini, Ramon A. Alvarez-Puebla

**Affiliations:** 1 Department of Physical and Inorganic Chemistry and EMaS, Universitat Rovira I Virgili, Carrer de Marcel.lí Domingo s/n, 43007 Tarragona, Spain; 2 ICREA, Passeig Lluís Companys 23, 08010 Barcelona, Spain

**Keywords:** cancer, diagnosis, prognosis, optical sensors, nanoparticles, SERS, circulating tumor cells, circulating tumor nucleic acids, oncoproteins

## Abstract

As medicine continues to advance our understanding of and knowledge about the complex and multifactorial nature of cancer, new major technological challenges have emerged in the design of analytical methods capable of characterizing and assessing the dynamic heterogeneity of cancer for diagnosis, prognosis and monitoring, as required by precision medicine. With this aim, novel nanotechnological approaches have been pursued and developed for overcoming intrinsic and current limitations of conventional methods in terms of rapidity, sensitivity, multiplicity, non-invasive procedures and cost. Eminently, a special focus has been put on their implementation in liquid biopsy analysis. Among optical nanosensors, those based on surface-enhanced Raman scattering (SERS) have been attracting tremendous attention due to the combination of the intrinsic prerogatives of the technique (e.g., sensitivity and structural specificity) and the high degree of refinement in nano-manufacturing, which translate into reliable and robust real-life applications. In this review, we categorize the diverse strategic approaches of SERS biosensors for targeting different classes of tumor biomarkers (cells, nucleic acids and proteins) by illustrating key recent research works. We will also discuss the current limitations and future research challenges to be addressed to improve the competitiveness of SERS over other methodologies in cancer medicine.

## 1. Introduction

The remarkable phenotypic, genetic and morphological heterogeneity of cancer poses critical challenges at precisely describing and characterizing the disease in a given current state. Thus, major efforts have been focused on the identification of larger panels of specific, sensitive and quantitative cancer biomarkers for improving diagnosis, prognosis, and risk stratification and predicting/monitoring the response to treatments [1]. Moreover, as single-biomarker tumor discrimination typically fails to provide an accurate classification of the disease as required by clinicians, the current focus on applied cancer research is the development of sensitive analytical tools for the rapid and cost-effective multidimensional characterization of a panel of biomarkers to yield a unique molecular signature of the malignancy [2,3].

In the last decade, nanotechnological approaches have emerged as promising tools to tackle new challenges in cancer research, most prominently the efficient translation of the more in-depth fundamental understanding of cancer biology into practical clinical applications [4,5]. Within the field of nanoscience, surface-enhanced Raman scattering (SERS) spectroscopy has gained prominence in biosensing [6,7,8,9]. In SERS, the excitation of localized surface plasmon resonances (LSPR) at the surface of nanostructured metals with light induces the massive intensification of the Raman scattering from molecules located in close proximity to the metallic surface. This effect yields an ultrasensitive plasmon-enhanced spectroscopic technique which retains the intrinsic structural specificity and experimental flexibility of Raman spectroscopy [10]. As impressive advances in instrumentation and nanofabrication techniques enabling the engineering of finely-tuned plasmonic nanomaterials continue, SERS is progressively expanding into the realm of viable biomedical applications. In cancer research, SERS is typically implemented in place of, or in combination with, fluorescence spectroscopy to address key limitations of such established technique. Most prominently, SERS sensing entails larger multiplexing capabilities for simultaneous quantitative interrogation at high imaging resolution (using a single excitation wavelength) as well as improved photostability, sensitivity and reduced tissue damages when employing near-infrared (NIR) lasers. On the other hand, it is worth highlighting that fluorescence imaging is intrinsically faster while currently maintaining a much larger availability of commercial products and established analytical protocols. 

In this review, we present the main strategic approaches undertaken for the implementation of SERS sensors in cancer diagnosis, prognosis and monitoring. We will illustrate relevant and recent examples while discussing key advantages and current limitations of the technique. Special focus is devoted to the application of SERS imaging to tumor tissues and identification of cancer-related materials in biological fluids (liquid biopsy).

## 2. SERS Imaging of Cancer Tissues and Single Cells

**Figure 1:**
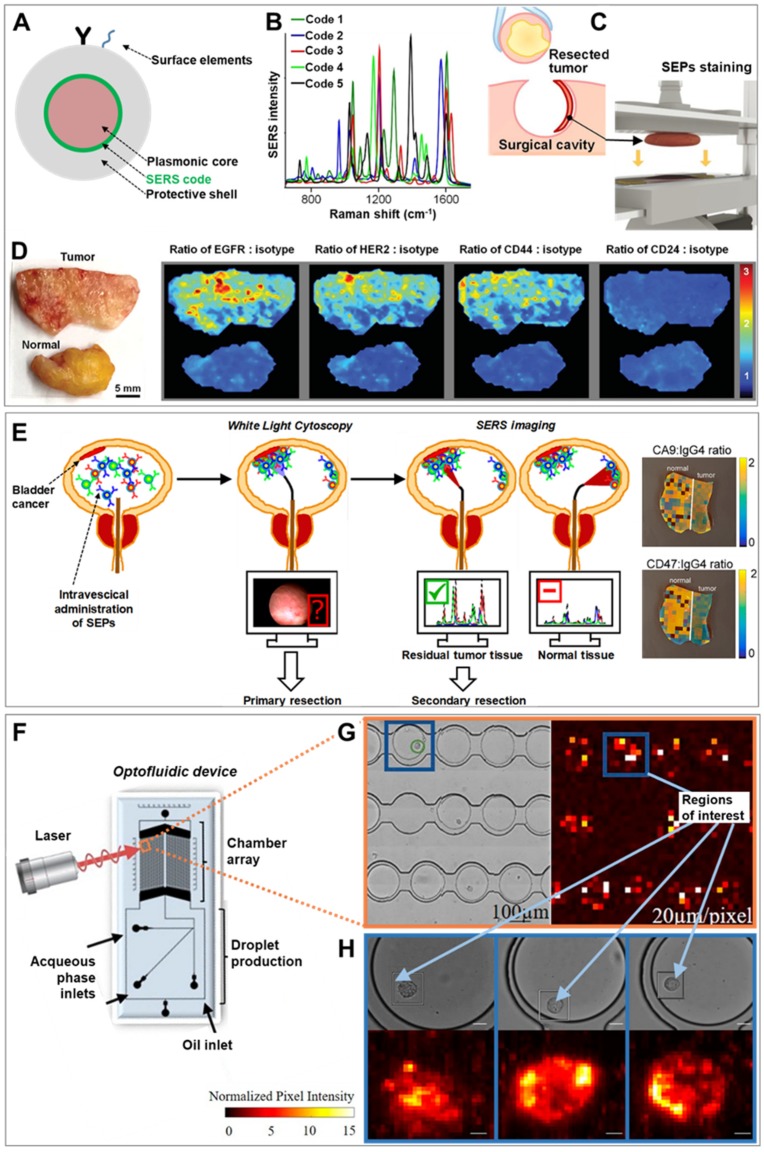
(**A**) Schematic depiction of the traditional surface-enhanced Raman scattering (SERS)-encoded nanoparticle (SEP) construction. (**B**–**D**) Application of SERS imaging to resected tumor tissue for intraoperative surgical guidance: (**B**) Unique SERS spectra of five different codes on gold particles coated with a silica shell. (**C**) Upon excision, the resected sample is placed in an automated staining device combining multiple quick dipping of the specimen into a SEPs solution with high-frequency vibration for fast and extensive topically applications of encoded particles onto the surface of the fresh tissue. (**D**) Ratiometric images biomarker targeting SEPs vs. negative control (isotype) obtained from raster-scanned SERS imaging (<3 min) of the illustrated human breast tumor and normal tissue. Adapted with permission from [18]. Copyright 2016, Wiley-VHC. (**E**) Schematic of the proposed application of intravesical SERS imaging for intraoperative endoscopic surgery. Adapted with permission from [19]. Copyright 2018, American Chemical Society. (**F**–**H**) SERS-microfluidic device for single live cell analysis: (**F**) Schematic depiction of the droplet-based optofluidic device; (**G**) Low-resolution map of the chamber array, (**H**) High-resolution map of individual cells encapsulated in droplets. Adapted with permission from [20] Copyright 2018, American Chemical Society.

The most straightforward and widely investigated implementation of SERS to cancer-related applications is as an optical imaging technique for molecular characterization of tumor tissues in place of (or in combination with) fluorescence spectroscopy. SERS-encoded particles (SEPs) may substitute for fluorescent reporters [11,12,13] (typically, fluorescently-labelled antibodies selective for specific protein receptors on the cell surface) as contrast agents. SEPs always combine a SERS molecular code, yielding a unique vibrational fingerprint (signal read-out), bound to a plasmonic core (mostly, silver or gold), as required for the enhancement of the weak Raman signal (Figure 1A). Additionally, an external protective layer (mainly, silica or polymeric) can be engineered around the encoded particles to improve stability while providing an optimal anchoring surface for further functionalization [14,15,16]. In fact, surface elements are typically further integrated on such outer shell to generate convenient physicochemical properties, such as selective targeting via functional molecules (e.g., antibodies, aptamers, folates etc.) [9,16,17]. 

The optical efficiency of SEPs is strictly related to the geometric features of the plasmonic core (particle compositional nature, size and shape, as well as interparticle interactions) [21,22]. Broadly speaking, the upper limit to the signal enhancement boosts as the nanoparticle is reshaped from spherical to anisotropic shapes (e.g., nanorods or nanostars) and, beyond, to nanoparticle aggregates. Concurrently, however, increasingly complicated practical challenges are posed for generating a broad panel of SEPs with reproducible and homogenous responses at a sufficient scale. In general, reliable and reproducible fabrication of nanomaterials in large quantities via standardized procedures still represents a bottleneck for implementation of nanomedicine. This explains why individual spherical nanoparticle cores remain the main preferred choice as optical enhancers in SEP-based applications (Table S1 provides a list of diverse plasmonic core geometries from SEPs employed in cancer-related research). We refer the interested readers to excellent reviews [14,15,16,17] for a more in-depth discussion on SEP fabrication, properties and applications.

The toxicity of nanomaterials is directly correlated with their retention and clearance. In the case of noble metal nanoparticles, a rapid and efficient excretion from the body is observed by reducing the nanoparticle size below the threshold for renal clearance (i.e., ca. < 5 nm) [23]. Interestingly, biodegradable assemblies of ultrasmall gold nanoparticles have shown the possibility of tuning the plasmonic response of the nanomaterial in the NIR region while avoiding long-time persistence in the body [24]. However, larger particle sizes are required to sustain LSPRs affording adequate electromagnetic enhancements. Thus, SEPs are characterized by relatively large intrinsic hydrodynamic diameters (typically > 100 nm) which currently prevents the clinical translation of SERS imaging of tumor tissues exploiting systemic routes of administration (e.g., intravenous injection) due to the major accumulation of metal particles in the body. Thus, major efforts have been recently focused on applications where SEPs can be directly delivered onto accessible tissues (e.g., via topical administration or direct intratumoral injection). Such approach drastically reduces the amount of nanomaterials required for the analysis thereby making the systemic uptake of the particles negligible [9,25,26,27,28].

In this regard, a remarkable example has been recently reported by Wang et al. [18] who developed a topical-staining protocol for rapid and quantitative multiplex SERS imaging of cancer biomarkers at the surfaces of freshly resected tissues to be used as an intraoperative tool for surgical guidance. Complete removal of a tumor at the primary site is a major issue in surgical oncology, as residual malignant microscopic foci may increase both the risk of recurrence and the rate of re-excision surgeries [29]. As a result, tumor removal is typically performed resecting also an area of surrounding normal tissue which, however, may compromise patient health and cosmesis. Current intraoperative pathology examination of frozen-sections fails to provide the required level of specificity, reliability and speediness, and a complete assessment for the presence of residual tumor can only be performed postoperatively. To tackle this problem, the authors prepared five differently SEPs, all consisting of a 60 nm spherical gold core, functionalized with a specific SERS code yielding a unique vibrational fingerprint (Figure 1B), and coated with a 30 nm silica layer. Monoclonal antibodies recognizing surface biomarkers overexpressed by breast cancer cells (EGFR, HER2, CD44 and CD24) were separately conjugated on four different SEPs. The last batch of contrast agents was functionalized with an antibody that does not actively target human proteins (IgG1, isotype control). Fast (<5 min) and extensive binding of SEPs at the surface of fresh human breast cancer tissues was achieved by repeatedly dipping the tissue into a solution of all contrast agents followed by high-frequency mechanical vibration (Figure 1C). Specimen were then rapidly imaged yielding, upon spectral deconvolution, ratiometric SERS maps depicting the intensity ratio of each of the four actively targeting SEPs against the background of non-targeted particles (Figure 1D). These maps precisely quantify the biomarker distributions, in agreement with immunohistochemistry data. Importantly, the use of the ratiometric approach, rather than the simple imaging of the absolute SERS signals, is required to address uneven and non-specific particle binding, removing then the need for extensive washing procedures. Following the same rationale, in vivo SERS imaging has been successfully applied in mice bearing ovarian adenocarcinoma on the peritoneum by Oseledchyk et al. [28]. A mixture of one tumor targeting and one non-targeting SEPs were directly injected in the peritoneal cavity, enabling the acquisition of radiometric images that accurately identify the location of tumor lesions in the tissue.

A particularly promising exploitation of SERS imaging is via Raman endoscopy for minimally invasive real-time molecular characterization of the surface of hollow organs. Currently, the “gold standard” for endoscopic procedures (white-light endoscopy, WLE) suffers from major limitations at detecting small tumor lesions, therefore requiring postoperative histological examinations to establish clinical decisions [30]. Notably, conventional clinical endoscopes can be suitably integrated with optical fibers for remote optical imaging and additional channels for local particle administration [27,31]. As a recent example, the feasibility of this approach has been demonstrated for multiplex imaging of bladder cancer tissue during transurethral resection [19]. Gambhir and co-workers administered ex-vivo in human bladders a cocktail of three antibody-conjugated SEPs (two actively targeting surface protein biomarkers CA9 and CD47, and one as control), acquiring ratiometric SERS via a Raman endoscope that yield accurate classification of tumor vs normal tissue. The proposed clinical application is outlined in Figure 1E. Upon pre-administration of SEPs into the patient bladder, transurethral cancer removal is carried out assisted by WLE. Subsequently, SERS mapping is performed using a Raman endoscope on resection margins and ambiguous regions on the white light image to provide accurate guidance for additional excisions when required.

In terms of multiplicity, the early work by Zavaleta et al. [32] remains one the most relevant example of multiplexed molecular imaging of cancer tissues with 10 different simultaneously discriminated SEP fingerprint. Nonetheless, the continuous development of sophisticated multivariate analysis techniques [33] is enabling access to the complex molecular information contained in multidimensional datasets. At the same time, the database of potential SERS codes is progressively expanding, with the design of novel classes of superior molecular reporters with reduced spectral overlapping [34] as well as the improvement of synthetic protocols for scalable fabrication of SEPs independently of the chemical nature of the SERS code [35,36]. Thus, we expect that several tens of SEPs can be soon routinely implemented in multiplex imaging analysis.

The development of advanced instrumentation focusing at reducing the acquisition time for Raman mapping is currently progressing [37]. However, scanning large areas of healthy tissues for identification of tumor spots with high spatial resolution still remains a major obstacle for the practical implementation of SERS imaging in the clinical practice. A relatively straightforward solution to such a low-throughput issue is offered by the designing of multimodal optical probe integrating fluorescent emitters into SEPs [38,39,40]. For instance, covalent loading of organic fluorophores can be easily achieved within the silica matrix surrounding the plasmonic core [41]. In this dual mode approach, fluorescence spectroscopy provides a first read-out for fast screening over large tissue areas while multiplexed SERS analysis is subsequently performed to precisely and quantitatively interrogate selected areas of interest.

Beyond imaging of tissues for discrimination of tumor vs healthy sites, SERS imaging with SEPs can be further extended to the analysis at the single cell level. In this scenario, the high multiplexing capability of SERS has the potential to provide accurate molecular phenotyping of the individual cancer cells. This entails the multidimensional characterization and discrimination of functionally relevant discrete cell sub-populations which are otherwise lost in population-averaged bulk measurements or not captured by genotypic studies. Notably, from static multiplex phenotyping of fixed samples [42], SERS single-cell analysis is advancing towards dynamic, non-destructive interrogation of live cells. Real-time monitoring of single cancer cell molecular diversity, under different conditions and extracellular stimuli, is expected to address fundamental questions about tumor biology while helping at expanding the library of clinically relevant biomarkers for better diagnosis, risk stratification for treatment decisions, prognosis, and drug development [1]. To this end, SERS spectroscopy is typically coupled with microfluidic technologies to facilitate single-cell manipulation and automated analysis [43]. In particular, trapping methods that avoid physical contacts or application of forces that perturb the biological nature of the cell are more suited for dynamic interrogations of molecular biomarkers [44]. An interesting combination of SERS and single-cell microfluidics has been recently reported by Willner et al. [20]. A droplet microfluidic device has been designed to encapsulate single-prostate cancer cells, previously incubated with SEPs targeting sialic acid expressed on the cancerous cell membrane, and store them into a chamber array for subsequent optical interrogation (Figure 1F). A data processing and analysis tool was developed to automatically perform and statistically analyze SERS mapping with tunable spectral resolution. Conceptually, the optical imaging is carried out in two steps. First, a large area of the chamber arrays is scanned with a low spectral resolution to rapidly identify areas of interest (Figure 1G). In a second step, these selected spots are mapped at a higher spectral resolution, thereby permitting the dynamic monitoring of sialic acid distribution at the single-cell level in a higher throughput fashion (Figure 1H). 

The potential use of multiplex SERS imaging in dynamic single cell analysis is an extremely promising route for acquiring new insights into the mechanisms driving heterogeneity of cancer cells in tumors and the identification of new biomarkers. Nonetheless, it remains far less explored as compared to the identification and quantification of circulating tumor cells (CTCs), a topic that will be discussed in the next section.

## 3. SERS Sensing of Tumor Biomarkers in Body Fluids (Liquid Biopsy)

Molecular and morphological characterization of solid tumor tissues acquired via traditional biopsies suffers from intrinsic limitations that hamper the advance of precision and personalized oncology. Besides their invasiveness, tissue biopsies are not appropriate for early diagnosis, capture only a snapshot of the tumor heterogeneity, and the accurate temporal monitoring of the disease evolution is typically unfeasible [45,46].

Non-invasive and rapid analysis of new sensitive and specific cancer biomarkers contained in biological fluids (i.e., liquid biopsy) has recently demonstrated the potential to address the limitations of traditional biopsy. The low abundance and chemical complexity of these novel biomolecular targets, including tumor-derived materials such circulating tumor cells (CTCs), circulating nucleic acids, protein and exosomes, make ultrasensitive and multiplexed SERS-based sensors particularly convenient for their analysis.

### 3.1. Circulating Tumor Cells (CTCs)

Differently to the single cell SERS imaging illustrated in Figure 1F–H, which aimed at the detailed dynamic molecular phenotypic characterization of single cells, simple identification and enumeration of cancer cells from a population of healthy cells prioritizes an experimental set-up favoring a reduction of the acquisition time over spatial resolution. For instance, Moskovits and co-workers [47] designed a microfluidic SERS device where cancerous and normal prostate cells, previously labelled with two targeting SEPs, are hydrodynamically focused on a single line flow through a channel and individually interrogated “on the fly” by Raman. While very intriguing, this approach is limited by the intrinsic acquisition time for current SERS measurements (>10 ms) which makes it unfeasible for clinical applications due to the extremely low-throughput screening of real samples. In fact, CTCs in the blood are rare events (usually, less than 10 CTCs per mL) among the vast background of billions of other cells [48]. It can be argued that, similarly to the strategy applied for improving the speed of imaging of tumor tissues, integration of dual fluorescence and SERS optical interrogation with microfluidics can offer an effective strategic solution to this problem. Once more, fast fluorescence read-out would be exploited for the initial screening and sorting of a large ensemble of CTCs while subsequent multiplexed characterization of different subpopulations within this selected fraction of cancer cells would be carried out via SERS. 

To tackle the low-abundance issue of CTCs, pre-isolation and enrichment procedures have been commonly applied prior to the molecular analysis by exploiting the diverse physical (e.g., size, mechanical properties, electric charge) or biomolecular (e.g., surface protein expression) characteristics of cancer cells [49]. For instance, Cell Search^®^ (Menarini Silicon Biosystems, Bologna, Italy), the only U.S. Food and Drug Administration (FDA) approved method for enumerating CTCs in the peripheral blood of patients with breast, colorectal, or prostate cancer, relies on the specific antigen-antibody recognition of epithelial cell adhesion molecule (EpCAM) and magnetic-based separation for CTC enrichment. The rationale for such an approach is the assumption that all cells in the blood expressing epithelial features (EpCAM-positive) and not expressing pan-leukocyte antigens (CD45-negative) are cancer cells. However, cancer research continues to reveal new classes of biologically relevant CTCs with high phenotypic variability and physical heterogeneous features, such as circulating cancer cell hybrids incorporating both hematopoietic and epithelial properties (EpCAM-positive, CD45-positive) [50]. Thus, it becomes evident that enrichment procedures based on just one parameter are most likely to disregard subpopulations of circulating cells equipped with potential metastatic capacity. 

**Figure 2:**
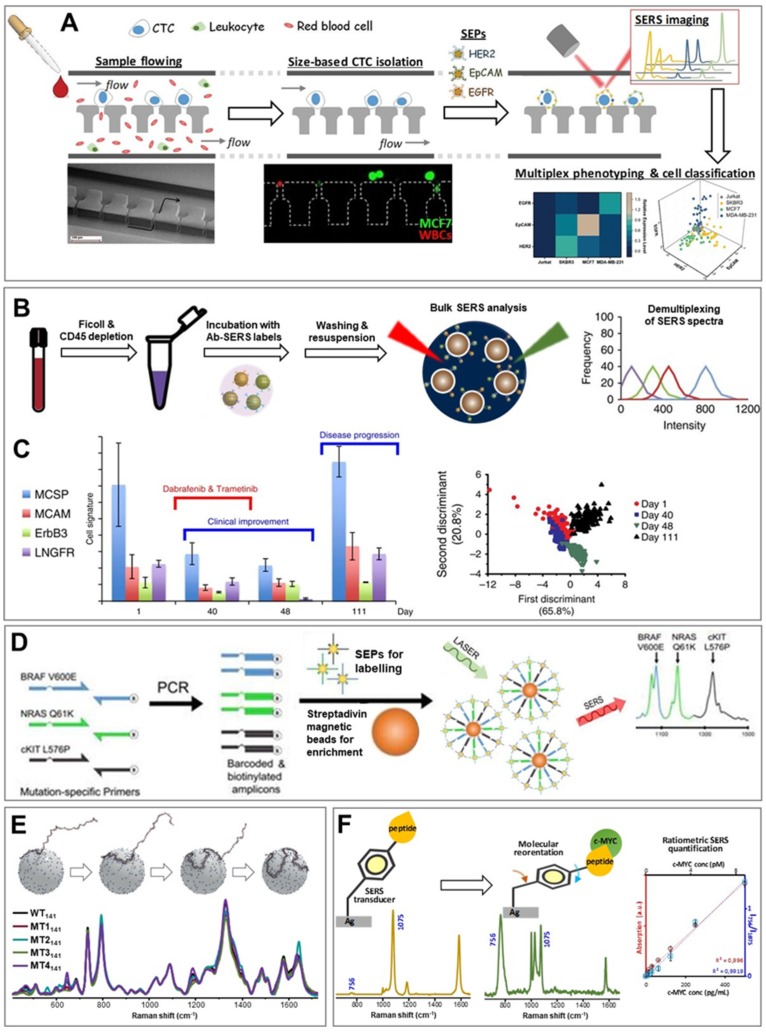
(**A**) Size-based capturing of circulating-tumor cells (CTCs) from blood, and multiplexed SERS phenotyping and chemometric classification of single cancer cells. (i) CTCs are isolated by flowing the whole blood through a nanogap array: see SEM image, arrow indicates the flow direction, and fluorescence image of fluorescently labelled individual cells (green, breast cancer cells MCF7; red, white blood cells) trapped at the edge of the pillar structure (dash lines show the edge of the pillar structure). (ii) Incubation with aptamer-SEPs enables the acquisition of SERS spectra that, upon deconvolution, yield single-cell SERS phenotypic signatures. Adapted with permission from ref. [54]. Copyright 2018, Wiley-VHC. (**B**,**C**) Real-time evolution of the phenotypic distribution of different cancer cell subpopulations: (**B**) Schematic outline of the experimental workflow. The ensemble of CTCs labelled with antibody-SEPs is interrogated via average analysis. Demultiplexing of the acquired SERS spectra is performed to yield SERS intensities from each code plotted as a frequency distribution curve. (**C**) Phenotypic signatures of CTCs in response to treatment. Patient with stage IV melanoma (day 1) was treated with dabrafenib and trametinib. After 1 month, treatment was discontinued due to toxicity. Cluster analysis of the SERS data is also reported. Adapted from Tsao et al., Nature Communications, 2019, 1482; DOI: 10.1038/s41467-018-03725-8 [55], licensed under CC BY 4.0. (**D**) PCR-SERS integration for identification of tumor DNAs using SEPs. Biotin-labelled amplicons of tumor DNA are recognized by SEPs and, subsequently, separated from the media with streptavidin-coated magnetic beads prior to multiplex SERS analysis. Adapted from Wee et al., Theranostics, 2016, 6, 1506; DOI: 10.7150/thno.15871 [56], licensed under CC BY-NC [56]. (**E**) Conformational classification of K-Ras point mutations (MT1-5) in long single-stranded DNAs via direct SERS analysis. Adapted with permission from [57]. Copyright 2017, WILEY-VCH. (**F**) Detection of the oncoprotein c-MYC in biological fluids using peptidic receptor derivatized with a SERS transducer which undergoes structural rearrangement upon target binding. The resulting spectral alterations, expressed as ratiometric intensities of two spectral marker bands at 756 and 1075 cm^−1^, are directly correlated with the c-MYC concent. Adapted with permission from [58]. Copyright 2016, American Chemical Society.

SERS spectroscopy has been typically integrated with various separation techniques for multiplexed CTC recognition and quantification in place of, as for tumor tissue imaging, fluorescence immunolabelling approaches for improved multiplexed phenotyping [51,52,53,54]. For instance, Wang et al. [54] combined SERS imaging with a microfluidic chip for size-based isolation of CTCs (13–25 µm diameter) from leukocytes and red blood cells (<11 µm), as illustrated in Figure 2A. A mixture of breast cancer cell lines (SKBR3, MCF7 and MDA-MB-231) belonging to the three major breast tumor subgroups (luminal, HER2 and basal) were spiked in human blood and flowed through a sieving chip with 12 µm gaps. Capturing of CTCs occurs with negligible retention of white blood cells. A cocktail of three SEPs conjugated with aptamers selectively targeting differently expressed surface biomarkers (HER2, EpCAM and EGFR) was subsequently injected and, upon surface binding, SERS spectral patterns from individual cells were generated. SERS data were finally demultiplexed using multivariate analytical methods to yield the weighted contribution from each SEPs and, thus, statistically categorize CTCs.

Multidimensional phenotyping of CTCs at the single cell level plays a central role in diagnosis, identification of therapeutic targets and resistance mechanisms and prognosis. On the other hand, averaged analysis providing phenotypic signatures of the whole CTC ensemble are more suitable for real-time monitoring of patient treatments and optimizing therapeutic interventions [59]. In a recent work, Tsao et al. [55] applied SERS analysis with SEPs for real-time monitoring of the collective phenotypic variation of CTCs isolated from blood samples of 10 stage-IV melanoma patients during therapy. The working principle of their method is outlined in Figure 2B. Blood samples were firstly depleted of red blood cells and peripheral blood mononuclear cells. The remaining cells were suspended and incubated with a mixture of five SEPs functionalized with four specific melanoma surface marker antibodies and one with an antibody isotype as a control. Upon removal of unbound SEPs via multiple washing steps, the cells were redispersed in a small volume of buffer and 150 SERS measurements were collected using a low magnification objective to yield spectra statistically representative of the entire illuminated scattering volume during the acquisition time (cells are in continuous Brownian motions within the sample). Deconvolution of each SERS spectra provides the individual code contribution to the overall signal. On the other hand, the frequency distribution of these code intensities across all measured events informs about the extent of dissimilarity between different biomarker expression levels. This is because each population of cancer cells is characterized by a specific relative content of surface biomarkers. Thus, the comprehensive absolute intensity (i.e., overall SEP concentration) is primarily informative about the number of CTCs (i.e., tumor progression) whereas the signal distribution width (i.e., the variability of relative SEP content from measurement to measurement) describes the extent of cell diversity. Figure 2C illustrates the data obtained from blood samples of a patient during drug treatment. CTC signatures display an overall drop of signal intensities associated with an efficient treatment (day 40) which, however, are recovered upon discontinuation caused by toxicity (day 111). The authors also observed a narrower signal distribution during treatment, indicating a reduction in CTC phenotypic diversity associated with selective elimination of drug-sensitive cancer cells. 

### 3.2. Secreted Cancer Biomarkers: Circulating Tumor Nucleic Acids, Proteins and Exosomes

Tumour-related biomolecules are released upon cancer cell death in the bloodstream generating a broad set of cell-free biomarkers that are proven extremely valuable for early-stage detection, prognosis and disease monitoring [60,61]. A well-known molecular marker originating from cancer cells is circulating tumor DNA (ctDNA), which provides direct information of the genetic alterations and mutations present in the solid tumor. Another clinically relevant class of nucleic acid material is microRNA (microRNAs), consisting of short single-stranded chains (18–25 nucleotides) that play a central role in gene regulation and whose altered expression levels have been related to cancer initiation and progression [62]. As compared to ctDNA, characterized by short half-life in the bloodstream, miRNAs display much higher stability.

Detection of nucleic acids by SERS has been extensively investigated, developing multiple approaches and modalities for ultrasensitive and quantitative analysis [63,64]. Within the frame of circulating nucleic acids sensing, a major technical problem is imposed by the very low abundance of cell-free cancer related material which, thus, demands extremely high quality isolation and, typically, integration of a variety of amplification strategies (e.g., polymerase-chain-reaction PCR, enzyme-based amplification, etc.) with the analytical method to afford the required level of sensitivity. The implementation of all these analytical steps, which also include collection and storage of unstable biomarkers, poses major obstacles to the robustness and reliability of the sensing platform [65]. In recent years, downstream SERS sensing of nucleic acids has been often integrated with PCR for detecting mutations in ctDNA [56,66,67], by profiting from the relatively simple and standardized PCR approaches for selectively amplify low abundance strands. For instance, Wee et al. [56] combined PCR amplification of ctDNA from plasma with SEPs-based imaging and magnetic bead-based enrichment for the simultaneous identification of clinically relevant point mutations in melanoma (Figure 2D). Amplification of tumor DNA is performed to generate PCR samples amplicons comprising a biotin molecule on one extreme and a 5′ overhang barcode sequence on the other. SEPs conjugated with oligonucleotides complementary to the barcode segment are incubated with the PCR samples, leading to the formation of a SEP/amplicon-biotin complex. This ensemble can be selectively isolated with a magnet upon binding with streptavidin-coated magnetic beads and, then, interrogated by the laser. The appearance in the vibrational spectra of SERS code marker bands informs about the occurrence of a specific mutation (with a limit of detection down to 10 mutant alleles, corresponding to 0.1% of the total DNA). 

Removal of the selected sequence from the complex biological matrix and its selective exponential amplification via PCR pave also the way for the effective implementation of direct SERS sensing approaches. In direct SERS, the analyte identification relies on the acquisition of its intrinsic vibrational spectrum upon adhesion onto the plasmonic surface. Thus, the target analyte is required to provide distinguishable SERS signal while co-adsorption by other background components has to be minimized to avoid significant signal overlapping. In this regard, spermine-coated silver colloids (AgSp) have been successfully exploited as an efficient plasmonic substrate to yield intense and reproducible SERS spectra of nucleic acid at low concentrations [63]. Positively-charged spermine molecules anchored onto the nanoparticle surface promote the rapid electrostatic binding of DNA via its negatively charged phosphate backbone and, consequently, the formation of DNA-mediated nanoparticle clusters where the biomolecules are entrapped at closely-spaced interparticle junctions (i.e., plasmonic hot-spots) [68]. Bulk SERS analysis of these colloidal suspensions generates SERS spectra that are an accurate representation of the specific composition and conformation of the nucleic acids in the sample. Notably, our group demonstrated such high molecular discrimination capabilities in the classification of clinically relevant point mutations within large K-Ras oncogene fragments (141 nucleotides) by spectrally discerning the large conformational changes that single- and multiple-base substitutions determine on long single-stranded chains (Figure 2E) [57]. Trau and coworkers [67] extended this approach to human sample interrogation by developing a nanodiagnostic assay for prostate cancer risk stratification. Total RNA is extracted from patient urines and parallel isothermal reverse transcription−recombinase polymerase amplification (RT-RPA) is applied to three clinically relevant RNA targets to yield double-stranded DNA amplicons that, upon purifications, are combined with AgSp and interrogated by direct SERS. Chemometric analysis of the SERS data statistically differentiate each amplicon signals with high clinical sensitivity and specificity.

Dysregulated expression of proteins, including expression of defect proteins, is also a hallmark of cancer. Remarkably, protein levels distribution provide a wealth of potentially accurate information for early and precise cancer diagnosis and prognosis [2]. In a similar fashion as for other cancer biomarkers, various strategies for SERS analysis has been applied for detection of multiple proteins exploiting SEPs as labelling agents and, mainly, antibody/aptamers as surface elements for selective target capturing [52]. In addition to these approaches, a novel hybrid SERS sensing scheme is progressively emerging as an efficient strategy for ultrasensitive quantification of proteins in complex biological matrices by profiting from the large target molecular size [58,69,70,71,72]. Here, the surface recognition elements are derivatized with a SERS-active moiety that firmly binds the plasmonic surface and acts as an efficient signal transducer by yielding an intense vibrational signature. Subsequent formation of the surface ligand/target protein complex results in the structural and/or conformational perturbation of the SERS transducer, as revealed by alterations of the spectral profile which can be quantitatively correlated with the number of recognition events. A major advantage of this sensing scheme over those based on SEPs is, therefore, the possibility of using the SERS-active surface element itself as the internal standard. For instance, our group synthesized a selective peptide receptor bearing a mercaptobenzoyl terminal unit for rapid and ultrasensitive quantification of the c-MYC oncoprotein in real human samples [58]. The SERS transducer moiety provides an intense vibrational signature that is drastically reshaped upon c-MYC binding as a result of the reorientation of the benzoyl ring over the plasmonic surface, in agreement with SERS surface selection rules. (Figure 2F). The ratiometric peak intensity of the band at 756 cm^−1^ (out-of-plane ring mode) vs. 1075 cm^−1^ (in-plane ring breathing) scales linearly with the c-MYC concentration.

Finally, exosomes are increasingly becoming the center of interest for their exploitation both as circulating cancer biomarkers and as enriched sources (most notably, miRNAs) of tumor markers in liquid biopsy [73]. Exosomes are nanosized extracellular vesicles (20–150 nm) playing a key role in cell-cell communications. Produced in the endosomal compartment and secreted into body fluids by most eukaryotic cells, these cell-derived materials display a composition (i.e., surface proteins distribution on their membrane as well as their cytosolic content) that reflects the state of the originating cells and their microenvironment [73]. Cancer-cell derived exosomes hold great potential over more established tumor biomarkers such as CTCs and ctDNA for multiple reasons, including larger availability in body fluids, biological stability in circulation, ease of separation, handling and characterization [73]. As a more in-depth understanding on the role of exosomes in tumorigenesis and their characterization as clinically relevant biomarkers is gained, SERS-based methods are progressively integrated as analytical tools by adapting those sensing strategies already illustrated for CTCs and other circulating tumor biomarkers [74,75,76].

## 4. Conclusions and Outlook

In summary, SERS has demonstrated its tremendous potential as a biomedical analytical tool for tackling the increasingly diverse and complex challenges of cancer research. Still, translation of SERS sensing into the clinic remains to be seen and, while developing more robust and reliable methodologies, researchers in this field currently faces critical obstacles that slow such implementation. Firstly, sensitive and quantitative SERS analysis requires, in the first instance, the fabrication of robust and highly efficient plasmonic substrates. However, as in the case of SEPs, modest enhancers like individual spherical gold particles have been largely preferred so far for their ease of production, scalability, homogenous response (i.e., for quantitative analysis) and commercial availability. Thus, to fully translate the ultrasensitive SERS detection of low abundant targets in complex biomatrices, scalable and simple nanomanufacturing protocols to produce higher performance SERS substrates have to come to fruition. Similarly, as the selective recognition of cancer biomarkers largely relies on indirect SERS approaches, we expect that an increase in commercial availability of synthetic ligands such as oligonucleotide/peptide aptamers will improve reproducibility and stability as well as lower the costs as compared to antibody-based sensing. Moreover, the potentially unlimited multiplexity of SERS has been largely restricted to proof-of-concepts studies employing few (4–5) codes. In addition to the development of enhanced chemometric tools for multidimensional SERS data analysis, the current extension of the SERS code library via rational design of synthetic, highly Raman active molecules will further facilitate larger multiplex sensing. Similarly, most of the current SERS studies have been carried out using sophisticated, expensive instruments or tailor-made systems, while clinical setting favors cost-efficient and manageable equipment for routine analysis. Accordingly, current efforts in applied research are focused on the design of cheaper, easy-to-use, portable Raman spectrometers for fast data acquisition. Instrument development is also called to efficiently integrate SERS sensing and Raman components with other techniques into multifunctional platforms, a need that has been highlighted in multiple examples reported in this review. Combination with both sample handling techniques (e.g., microfluidics, magnetic separation) and other characterization tools (e.g., fluorescence spectroscopy) is required to address intrinsic limitations of SERS as a stand-alone technique, allowing for high-throughput measurements with more standardized and automated procedures as well as increased multidimensional information. 

## Supplementary Materials

The following are available online at https://www.mdpi.com/2072-6694/11/6/748/s1, Table S1: Examples of SEPs employed in cancer research classified according to the plasmonic core geometry.
