# Supplementary material for: Surface-Enhanced Raman Spectroscopy in Cancer Diagnosis, Prognosis and Monitoring
Source: Cancers (Basel). 2019 May 29;11(6):748. doi: 10.3390/cancers11060748 (PMC6627759; doi:10.3390/cancers11060748)
Supplement: Supplementary file 1 [file cancers-11-00748-s001.pdf]

Supplementary Materials: Surface-Enhanced Raman Spectroscopy in Cancer Diagnosis, Prognosis and Monitoring

Luca Guerrini and Ramon A. Alvarez-Puebla

Table S1. Examples of SEPs employed in cancer research classified according to the plasmonic core geometry (NP = nanoparticle).

Plasmonic Nanostructure	References
Nanospheres	Gold, refs.[1–28]; and Silver, ref. [29]
Core@shell nanospheres	[30,31]
Hollow nanospheres	[32]
Nanorods	[33-38]
Nanoprisms	[39]
Nanostars	[40–44]
Nanooctahedras	[45]
NP clusters	[46–48]
NP decorated microbeads	[49,50]

References

1. Wang, Y.; Kang, S.; Khan, A.; Ruttner, G.; Leigh, S.Y.; Murray, M.; Abeytunge, S.; Peterson, G.; Rajadhyaksha, M.; Dintzis, S.; et al. Quantitative molecular phenotyping with topically applied SERS nanoparticles for intraoperative guidance of breast cancer lumpectomy. *Sci. Rep.* **2016**, *6*, 21242.
2. Mallia, R.J.; McVeigh, P.Z.; Fisher, C.J.; Veilleux, I.; Wilson, B.C. Wide-field multiplexed imaging of EGFR-targeted cancers using topical application of NIR SERS nanoprobes. *Nanomedicine* **2015**, *10*, 89–101.
3. Zavaleta, C.L.; Garai, E.; Liu, J.T.C.; Sensarn, S.; Mandella, M.J.; Van de Sompel, D.; Friedland, S.; Van Dam, J.; Contag, C.H.; Gambhir, S.S. A Raman-based endoscopic strategy for multiplexed molecular imaging. *Proc. Natl. Acad. Sci. USA* **2013**, *110*, E2288–E2297.
4. Zavaleta, C.L.; Smith, B.R.; Walton, I.; Doering, W.; Davis, G.; Shojaei, B.; Natan, M.J.; Gambhir, S.S. Multiplexed imaging of surface enhanced Raman scattering nanotags in living mice using noninvasive Raman spectroscopy. *Proc. Natl. Acad. Sci. USA* **2009**, *106*, 13511–13516.
5. Keren, S.; Zavaleta, C.; Cheng, Z.; de la Zerda, A.; Gheysens, O.; Gambhir, S.S. Noninvasive molecular imaging of small living subjects using Raman spectroscopy. *Proc. Natl. Acad. Sci. USA* **2008**, *105*, 5844–5849.
6. Jokerst, J.V.; Miao, Z.; Zavaleta, C.; Cheng, Z.; Gambhir, S.S. Affibody-Functionalized Gold–Silica Nanoparticles for Raman Molecular Imaging of the Epidermal Growth Factor Receptor. *Small* **2011**, *7*, 625–633.
7. Bohndiek, S.E.; Wagadarikar, A.; Zavaleta, C.L.; Van de Sompel, D.; Garai, E.; Jokerst, J.V.; Yazdanfar, S.; Gambhir, S.S. A small animal Raman instrument for rapid, wide-area, spectroscopic imaging. *Proc. Natl. Acad. Sci. USA* **2013**, *110*, 12408–12413.
8. Kircher, M.F.; de la Zerda, A.; Jokerst, J.V.; Zavaleta, C.L.; Kempen, P.J.; Mittra, E.; Pitter, K.; Huang, R.; Campos, C.; Habte, F.; et al. A brain tumor molecular imaging strategy using a new triple-modality MRI-photoacoustic-Raman nanoparticle. *Nat. Med.* **2012**, *18*, 829.
9. Wang, Y.W.; Doerksen, J.D.; Kang, S.; Walsh, D.; Yang, Q.; Hong, D.; Liu, J.T.C. Multiplexed Molecular Imaging of Fresh Tissue Surfaces Enabled by Convection-Enhanced Topical Staining with SERS-Coded Nanoparticles. *Small* **2016**, *12*, 5612–5621.
10. Davis, R.M.; Kiss, B.; Trivedi, D.R.; Metzner, T.J.; Liao, J.C.; Gambhir, S.S. Surface-Enhanced Raman Scattering Nanoparticles for Multiplexed Imaging of Bladder Cancer Tissue Permeability and Molecular Phenotype. *ACS Nano* **2018**, *12*, 9669–9679.
11. Yarbakht, M.; Nikkhah, M.; Moshaii, A.; Weber, K.; Matthäus, C.; Cialla-May, D.; Popp, J. Simultaneous isolation and detection of single breast cancer cells using surface-enhanced Raman spectroscopy. *Talanta* **2018**, *186*, 44–52.
12. Cho, H.Y.; Hossain, M.K.; Lee, J.H.; Han, J.; Lee, H.J.; Kim, K.J.; Kim, J.H.; Lee, K.B.; Choi, J.W. Selective isolation and noninvasive analysis of circulating cancer stem cells through Raman imaging. *Biosens. Bioelectron.* **2018**, *102*, 372–382.

13. Zhang, Y.; Wang, Z.; Wu, L.; Zong, S.; Yun, B.; Cui, Y. Combining Multiplex SERS Nanovectors and Multivariate Analysis for In Situ Profiling of Circulating Tumor Cell Phenotype Using a Microfluidic Chip. *Small* **2018**, *14*, 1704433.
14. Tsao, S.C.H.; Wang, J.; Wang, Y.; Behren, A.; Cebon, J.; Trau, M. Characterising the phenotypic evolution of circulating tumour cells during treatment. *Nat. Commun.* **2018**, *9*, 1482.
15. Wee, E.J.H.; Wang, Y.; Tsao, S.C.H.; Trau, M. Simple, sensitive and accurate multiplex detection of clinically important melanoma DNA mutations in circulating tumour DNA with SERS nanotags. *Theranostics* **2016**, *6*, 1506–1513.
16. Andreou, C.; Neuschmelting, V.; Tschaharganeh, D.-F.; Huang, C.-H.; Oseledchyk, A.; Iacono, P.; Karabeber, H.; Colen, R.R.; Mannelli, L.; Lowe, S.W.; et al. Imaging of Liver Tumors Using Surface-Enhanced Raman Scattering Nanoparticles. *ACS Nano* **2016**, *10*, 5015–5026.
17. Conde, J.; Bao, C.; Cui, D.; Baptista, P.V.; Tian, F. Antibody-drug gold nanoantennas with Raman spectroscopic fingerprints for in vivo tumour theranostics. *J. Control. Release* **2014**, *183*, 87–93.
18. Dinish, U.S.; Balasundaram, G.; Chang, Y.-T.; Olivo, M. Actively Targeted In Vivo Multiplex Detection of Intrinsic Cancer Biomarkers Using Biocompatible SERS Nanotags. *Sci. Rep.* **2014**, *4*, 4075.
19. Dinish, U.S.; Song, Z.; Ho, C.J.H.; Balasundaram, G.; Attia, A.B.E.; Lu, X.; Tang, B.Z.; Liu, B.; Olivo, M. Single Molecule with Dual Function on Nanogold: Biofunctionalized Construct for In Vivo Photoacoustic Imaging and SERS Biosensing. *Adv. Funct. Mater.* **2015**, *25*, 2316–2325.
20. Kang, S.; Wang, Y.; Reder, N.P.; Liu, J.T.C. Multiplexed Molecular Imaging of Biomarker-Targeted SERS Nanoparticles on Fresh Tissue Specimens with Channel-Compressed Spectrometry. *PLOS ONE* **2016**, *11*, e0163473.
21. Karabeber, H.; Huang, R.; Iacono, P.; Samii, J.M.; Pitter, K.; Holland, E.C.; Kircher, M.F. Guiding Brain Tumor Resection Using Surface-Enhanced Raman Scattering Nanoparticles and a Hand-Held Raman Scanner. *ACS Nano* **2014**, *8*, 9755–9766.
22. Maiti, K.K.; Dinish, U.S.; Samanta, A.; Vendrell, M.; Soh, K.S.; Park, S.J.; Olivo, M.; Chang, Y.T. Multiplex targeted in vivo cancer detection using sensitive near-infrared SERS nanotags. *Nano Today* **2012**, *7*, 85–93.
23. Qian, X.M.; Peng, X.H.; Ansari, D.O.; Yin-Goen, Q.; Chen, G.Z.; Shin, D.M.; Yang, L.; Young, A.N.; Wang, M.D.; Nie, S.M. In vivo tumor targeting and spectroscopic detection with surface-enhanced Raman nanoparticle tags. *Nat.Biotech.* **2008**, *26*, 83–90.
24. Wang, Y.W.; Khan, A.; Som, M.; Wang, D.; Chen, Y.; Leigh, S.Y.; Meza, D.; McVeigh, P.Z.; Wilson, B.C.; Liu, J.T.C. Rapid ratiometric biomarker detection with topically applied SERS nanoparticles. *Technology* **2014**, *02*, 118–132.
25. Bizzarri, A.R.; Moscetti, I.; Cannistraro, S. Surface enhanced Raman spectroscopy based immunosensor for ultrasensitive and selective detection of wild type p53 and mutant p53(R175H). *Anal. Chim. Acta* **2018**, *1029*, 86–96.
26. Li, M.; Wu, J.; Ma, M.; Feng, Z.; Mi, Z.; Rong, P.; Liu, D. Alkyne- and Nitrile-Anchored Gold Nanoparticles for Multiplex SERS Imaging of Biomarkers in Cancer Cells and Tissues. *Nanotheranostics* **2019**, *3*, 113–119.
27. Nicolson, F.; Jamieson, L.E.; Mabbott, S.; Plakas, K.; Shand, N.C.; Detty, M.R.; Graham, D.; Faulds, K. Through tissue imaging of a live breast cancer tumour model using handheld surface enhanced spatially offset resonance Raman spectroscopy (SESORRS). *Chem. Sci.* **2018**, *9*, 3788–3792.
28. Wang, Y.; Yang, Q.; Kang, S.Y.; Wall, M.A.; Liu, J.T.C. High-speed Raman-encoded molecular imaging of freshly excised tissue surfaces with topically applied SERRS nanoparticles. *J. Biomed. Opt.* **2018**, *23*, 8.
29. Zhao, P.; Li, H.X.; Li, D.W.; Hou, Y.J.; Mao, L.; Yang, M.; Wang, Y. A SERS nano-tag-based magnetic-separation strategy for highly sensitive immunoassay in unprocessed whole blood. *Talanta* **2019**, *198*, 527–533.
30. Hu, C.; Shen, J.; Yan, J.; Zhong, J.; Qin, W.; Liu, R.; Aldalbahi, A.; Zuo, X.; Song, S.; Fan, C.; et al. Highly narrow nanogap-containing Au@Au core–shell SERS nanoparticles: size-dependent Raman enhancement and applications in cancer cell imaging. *Nanoscale* **2016**, *8*, 2090–2096.
31. He, J.; Dong, J.; Hu, Y.; Li, G.; Hu, Y. Design of Raman tag-bridged core-shell Au@Cu 3 (BTC) 2 nanoparticles for Raman imaging and synergistic chemo-photothermal therapy. *Nanoscale* **2019**, *11*, 6089–6100.
32. Ju, K.-Y.; Lee, S.; Pyo, J.; Choo, J.; Lee, J.-K. Bio-inspired Development of a Dual-Mode Nanoprobe for MRI and Raman Imaging. *Small* **2015**, *11*, 84–89.

33. Chen, S.; Bao, C.; Zhang, C.; Yang, Y.; Wang, K.; Chikkaveeraiah, B.V.; Wang, Z.; Huang, X.; Pan, F.; Wang, K.; et al. EGFR antibody conjugated bimetallic Au@Ag nanorods for enhanced SERS-based tumor boundary identification, targeted photoacoustic imaging and photothermal therapy. *Nano. Biomed. Eng.* **2016**, *8*, 315–328.
34. Jokerst, J.V.; Cole, A.J.; Van de Sompel, D.; Gambhir, S.S. Gold Nanorods for Ovarian Cancer Detection with Photoacoustic Imaging and Resection Guidance via Raman Imaging in Living Mice. *ACS Nano* **2012**, *6*, 10366–10377.
35. Nima, Z.A.; Mahmood, M.; Xu, Y.; Mustafa, T.; Watanabe, F.; Nedosekin, D.A.; Juratli, M.A.; Fahmi, T.; Galanzha, E.I.; Nolan, J.P.; et al. Circulating tumor cell identification by functionalized silver-gold nanorods with multicolor, super-enhanced SERS and photothermal resonances. *Sci. Rep.* **2014**, *4*, 8.
36. Qian, J.; Jiang, L.; Cai, F.; Wang, D.; He, S. Fluorescence-surface enhanced Raman scattering co-functionalized gold nanorods as near-infrared probes for purely optical in vivo imaging. *Biomaterials* **2011**, *32*, 1601–1610.
37. Pang, Y.; Wang, C.; Xiao, R.; Sun, Z. Dual-Selective and Dual-Enhanced SERS Nanoprobes Strategy for Circulating Hepatocellular Carcinoma Cells Detection. *Chem. Eur. J.* **2018**, *24*, 7060–7067.
38. Jiang, L.; Qian, J.; Cai, F.H.; He, S.L. Raman reporter-coated gold nanorods and their applications in multimodal optical imaging of cancer cells. *Anal Bioanal Chem* **2011**, *400*, 2793–2800.
39. Ruan, H.; Wu, X.; Yang, C.; Li, Z.; Xia, Y.; Xue, T.; Shen, Z.; Wu, A. A Supersensitive CTC Analysis System Based on Triangular Silver Nanoprisms and SPION with Function of Capture, Enrichment, Detection, and Release. *ACS Biomater. Sci. Eng.* **2018**, *4*, 1073–1082.
40. Oseledchyk, A.; Andreou, C.; Wall, M.A.; Kircher, M.F. Folate-Targeted Surface-Enhanced Resonance Raman Scattering Nanoprobe Ratiometry for Detection of Microscopic Ovarian Cancer. *ACS Nano* **2017**, *11*, 1488–1497.
41. Song, C.; Li, F.; Guo, X.; Chen, W.; Dong, C.; Zhang, J.; Zhang, J.; Wang, L. Gold nanostars for cancer cell-targeted SERS-imaging and NIR light-triggered plasmonic photothermal therapy (PPTT) in the first and second biological windows. *J. Mater. Chem. B* **2019**, *7*, 2001–2008.
42. Bhamidipati, M.; Cho, H.Y.; Lee, K.B.; Fabris, L. SERS-Based Quantification of Biomarker Expression at the Single Cell Level Enabled by Gold Nanostars and Truncated Aptamers. *Bioconjugate Chem.* **2018**, *29*, 2970–2981.
43. Ou, Y.C.; Webb, J.A.; O'Brien, C.M.; Pence, I.J.; Lin, E.C.; Paul, E.P.; Cole, D.; Ou, S.H.; Lapierre-Landry, M.; DeLapp, R.C.; et al. Diagnosis of immunomarkers in vivo via multiplexed surface enhanced Raman spectroscopy with gold nanostars. *Nanoscale* **2018**, *10*, 13092–13105.
44. Liu, Y.; Yuan, H.K.; Fales, A.M.; Register, J.K.; Vo-Dinh, T. Multifunctional gold nanostars for molecular imaging and cancer therapy. *Front. Chem.* **2015**, *3*, 7.
45. Bodelón, G.; Montes-García, V.; Fernández-López, C.; Pastoriza-Santos, I.; Pérez-Juste, J.; Liz-Marzán, L.M. Au@pNIPAM SERRS Tags for Multiplex Immunophenotyping Cellular Receptors and Imaging Tumor Cells. *Small* **2015**, *11*, 4149–4157.
46. Pallaoro, A.; Hoonejani, M.R.; Braun, G.B.; Meinhart, C.D.; Moskovits, M. Rapid Identification by Surface-Enhanced Raman Spectroscopy of Cancer Cells at Low Concentrations Flowing in a Microfluidic Channel. *ACS Nano* **2015**, *9*, 4328–4336.
47. Zheng, Z.H.; Wu, L.; Li, L.; Zong, S.F.; Wang, Z.Y.; Cui, Y.P. Simultaneous and highly sensitive detection of multiple breast cancer biomarkers in real samples using a SERS microfluidic chip. *Talanta* **2018**, *188*, 507–515.
48. Wang, Z.; Wu, H.; Wang, C.; Xu, S.; Cui, Y. Gold aggregates- and quantum dots- embedded nanospheres: Switchable dual-mode image probes for living cells. *J. Mater. Chem.* **2011**, *21*, 4307–4313.
49. Jeong, S.; Kim, Y.-i.; Kang, H.; Kim, G.; Cha, M.G.; Chang, H.; Jung, K.O.; Kim, Y.-H.; Jun, B.-H.; Hwang, D.W.; et al. Fluorescence-Raman Dual Modal Endoscopic System for Multiplexed Molecular Diagnostics. *Sci. Rep.* **2015**, *5*, 9455.
50. Wang, Z.; Zong, S.; Chen, H.; Wang, C.; Xu, S.; Cui, Y. SERS-Fluorescence Joint Spectral Encoded Magnetic Nanoprobes for Multiplex Cancer Cell Separation. *Adv. Healthc. Mater.* **2014**, *3*, 1889–1897.

© 2019 by the authors. Licensee MDPI, Basel, Switzerland. This article is an open access article distributed under the terms and conditions of the Creative Commons Attribution (CC BY) license (http://creativecommons.org/licenses/by/4.0/).
